# Bioethical Decisions in Neonatal Intensive Care: Neonatologists’ Self-Reported Practices in Greek NICUs

**DOI:** 10.3390/ijerph17103465

**Published:** 2020-05-15

**Authors:** Maria Dagla, Vasiliki Petousi, Antonios Poulios

**Affiliations:** 1 Department of Midwifery, University of West Attica, 122 41 Athens, Greece; 2 Department of Sociology, University of Crete, 741 00 Rethymno, Greece; petousiv@uoc.gr; 3 Department of Psychology, National and Kapodistrian University of Athens, 157 72 Athens, Greece; antpls@yahoo.gr

**Keywords:** intensive care, NICU, ethical decision-making, withholding, withdrawing

## Abstract

This study presents, for the first time, empirical data on practices regarding bioethical decision-making in treatment of preterm and ill newborns in Greece. The aim of the study was to: (a) record self-reported practices and involvement of Greek physicians in decisions of withholding and withdrawing neonatal intensive care, and (b) explore the implication of cultural, ethical, and professional parameters in decision-making. **Methods:** 71 physicians, employed fulltime in all public Neonatal Intensive Care Units (NICUs) (n = 17) in Greece, completed an anonymous questionnaire between May 2009 and May 2011. **Results:** One-third of the physicians in our sample admitted that they have, at least once in the past, decided the limitation of intensive care of a newborn close to death (37.7%) and/or a newborn with unfavorable neurological prognosis (30.8%). The higher the physicians’ support towards the value of quality of human life, the more probable it was that they had taken a decision to withhold or withdraw neonatal intensive care (*p* < 0.05). **Conclusions:** Our research shows that Greek NICU physicians report considerably lower levels of ethical decision-making regarding preterm and ill newborns compared to their counterparts in other European countries. Clinical practices and attitudes towards ethical decision-making appear to be influenced mainly by the Greek physicians’ values.

## 1. Introduction

Preterm birth, birth before the 37th week or the 259th day of gestation [1], is a major perinatal problem with severe implications for public health. Compared to full-term newborns, preterm ones, especially those born before the 32nd week of pregnancy, face higher risk of dying after labour or developing serious neurodevelopmental disorders that will affect their short- or long-term quality of life [2,3,4,5,6,7,8,9]. Recent studies indicate some improvement in the survival rates of extremely preterm babies [2,3,10,11,12,13]. However, the rates of death remain very high [10,14], the neurodevelopmental outcomes of these newborns are relatively poor [15,16], and survival without impairment is substantially lower for children born at <25 weeks’ gestational age than for those born later [10].

Given the global increasing prevalence of preterm births [17,18] and especially of births of extremely preterm newborns (<28th week) and very preterm newborns (between 28th and 31st week) [19], the above epidemiological data raise significant concerns within the scientific community. Moreover, the success rate of intensive care practices to prevent death or the development of serious disabilities of these newborns is limited [10,14,15,16]. Thus, challenging bioethical questions, regarding the initiation and purpose of intensive care in these clinical cases, as well as the extent and limits of the relevant medical interventions are raised [20]: To what extent will the aggressive intensive care benefit the newborn born at <28 weeks’ gestational age? To what extent is the implementation of limits in interventional practices and therapies that keep these babies in life, ethically justified?

Such bioethical questions often emerge in Neonatal Intensive Care Units (NICUs) and are intensified due to continuous medical and technological advancements. International research shows that the majority of healthcare professionals who provide neonatal care have faced the bioethical dilemma: use complete, aggressive intensive care or place limits in the interventional medical practices that support the life of the newborn? [21]. Cuttini et al. [22] argue that a significant proportion of physicians in Europe have been involved in “ethical decisions” at least once during their career, even without becoming aware of it. Other empirical studies have also shown that healthcare professionals are involved in decisions regarding the limitation of intensive neonatal care in developed, as well as, in developing countries [23,24,25,26,27,28].

Ethical decisions have been defined as “decisions to withhold or withdraw life-sustaining treatment when the balance between the benefits and the burdens of intensive care is uncertain, or even clearly unfavorable” [29]. Such ethically charged clinical decisions that may lead to death or acceleration of death [30] are divided into three groups: (a) deliberate withholding or withdrawing of life-sustaining therapy or life support, e.g., not performing necessary cardiopulmonary resuscitation, not administering medication which is necessary for survival etc; (b) deliberate withdrawal of life support machine or therapeutic treatment; and (c) deliberate termination of life [31]. Regardless of whether they are officially recorded or whether they are legally permissible or not, these clinical practices in NICUs (withholding or withdrawing of life-sustaining treatment) are likely to occur in many countries [20,26,32,33,34].

International studies have shown that the attitude and practices of healthcare professionals regarding ethical decision-making in intensive neonatal care differs significantly depending on the specific legal, economic, religious, historical, and general cultural conditions and characteristics of each country [35,36,37,38]. Country of origin emerges as the main differentiation factor of clinical behaviors pertaining to neonatal intensive care [37,38,39,40]. At a broader level, differences are recorded between Northern and Southern European countries, as well as Latin American and Northern European countries [22,24,37,38,41,42]. Moreover, differences have been recorded within individual countries [39].

Given (a) the significant implications of the ethically charged clinical decisions concerning neonatal intensive care and (b) the observed differences between countries, the need for empirical studies documenting their frequency and the determinants of the relevant decision-making processes at the international and national level, becomes evident. The relative lack of such studies at the international level and thus, the need thereof, is documented in the literature [43]. This need, however, is particularly pronounced for countries such as Greece, which on the one hand lack an appropriate framework (e.g., legal provisions, national guidelines, recommendations, clinical protocols), and on the other, lack empirical data on physicians’ practices and ethical decision-making processes. With respect to the latter, in Greece, some research on limiting intensive care for adults is available [44,45,46,47]. However, no equivalent data are available for neonatologists’ practices and decision-making processes concerning intensive care for neonates.

This article aims at filling this gap through empirical data on ethical decision-making concerning intensive care for newborns in Greece. These data are part of a larger research project, conducted for the first time in Greece, with the aim of (a) assessing the ethical acceptability of clinical practices that set limits to the provision of neonatal intensive care; (b) investigating the factors that delineate the ethical acceptability of such limitations; and (c) providing an empirical basis for comparisons between Greece and other countries [48]. The purpose of the present article is: (a) to record self-reported practices and self-reported degree of involvement of Greek physicians at NICUs in decisions of withholding and withdrawing neonatal intensive care and (b) to investigate the impact of socio-cultural and ethical characteristics in the physicians’ ethical decision-making.

## 2. Materials and Methods

This study investigates aspects of the ethical decision-making process regarding provision of intensive care to babies born at the limits of human viability or suffering from a severe disease based on the internationally recognized research protocol of the EURONIC Project (“Parents’ Information and Ethical Decision Making in Neonatal Intensive Care Units: Staff Attitudes and Opinions”) [49]. The overall study’s objectives and methods have been presented in detail elsewhere [48,50]. The current article presents findings on physicians’ self-reported practices and their experience on ethical decision-making in clinical cases in which the infant (a) was near death and/or, (b) had a severe neurological prognosis.

The procedure for sample selection and the process of translation and cultural adaptation of the research tools used in the broader and the current study are detailed in Daglas & Petousi [48] and briefly presented here. All healthcare professionals (*n* = 495) who, during the time of the research (May 2009 until March 2011), were employed full time at the 17 NICUs operating in all public Greek hospitals and meeting the EURONIC inclusion criteria [48,49], were invited to participate. Of those, 251 healthcare professionals (98 midwives, 82 nurses and 71 physicians/neonatologists) completed the questionnaires. The response rate was 50.7% considered adequate for studies with printed, mailed questionnaires [51]. Physicians/neonatologists were asked to complete the “Questionnaire for medical staff”, while midwives and nurses the “Questionnaire for nursing staff’”. Both questionnaires were codified and self-administered. The questionnaires had been previously developed and used in the EURONIC project. They were translated and culturally adjusted to the Greek reality for the purposes of the current research [48]. Information regarding the framework, equipment and staff of NICUs was obtained from the “Unit Description Questionnaire”, a self-administered questionnaire, completed by the Director or Head of the NICU. In the current article we only report on the answers of the 71 physicians/neonatologists included in our sample.

Throughout the study, special attention was given to respecting the code of ethics of research, as already presented in another paper [48]. All necessary ethics approvals and permissions were granted: (a) by every Board of Directors and every Ethics Committee of all participating hospitals and (b) by the Directors of the participating NICUs. All participants signed consent forms after being fully informed orally and in writing about the purpose and the methodology of the study as well as the implemented procedures safeguarding the confidentiality of data.

Data were analyzed using SPSS software. The self-reported participation of Greek neonatologists in decisions regarding the limitation of intensive neonatal care was set as the dependent variable. At the single-factor analysis, the following were set as independent variables: (a) personal characteristics of physicians (gender, age, having had children, religiousness—defined as religion’s importance in one’s life (religiousness rather than religious affiliation was used in the current study given that in Greece, approximately 95% of people are Orthodox Christians)); (b) professional characteristics (length of experience in neonatal intensive care); (c) variables related to hospital (institution—university/public, location); (d) variables associated with NICU (number of hospitalized infants, number of monitors, total number of employees, total number of physicians, total number of midwives and nurses); and (e) the “attitude score”, an overall assessment of value assigned to the human life.

The “attitude score” was derived from participants’ answers to a set of seven statements using a five-point Likert scale (from 1 = “strongly agree” to 5 = “strongly disagree”) [50]. These statements come from a scale developed by Rebagliato et al. [34]. The scale is conceived as a continuum between the “pro-life approach”, that is, full alignment (full agreement) with the principle of the inherent (absolute) value of human life and the “the quality of life approach” or else full alignment with the principle that quality of life needs to be considered. Based on the numerical values assigned to the scale, the lower the “attitude score”, the closer the alignment with the “pro-life approach”, which holds that human life should be maintained by applying every possible technical and medical intervention, regardless of the outcome and the required cost. Inversely, the higher the “attitude score”, the closer the alignment with the “quality of life approach”, which holds that treatment should be decided on the basis of the prognosis for the long-term quality of life of the newborn.

In the single-factor analyses, the comparison of percentage ratios was made using the chi-square test, while the comparison of means was made using the independent samples t-test and the One-Way Analysis of Variance. Pearson’s r correlation was used for the calculation of the correlation self-reported participation of Greek physicians in decisions regarding the limitation of intensive neonatal care. Logistic regression analysis was used in the statistical model, since the dependent variable was categorical (yes/maybe/no, physicians having taken such decision). The model was approved based on the Hosmer and Lemeshow test. The Nagelkerke’s R^2^ value obtained was 0.35. The entire model was shown to contribute significantly to the prediction of the dependent variable (*p* < 0.05). The following were set as prediction variables: gender, age, length of experience in NICU, religiousness, having had children, and whether physicians agree or not with the participation of parents in ethical decision-making regarding their children and physicians’ attitude towards the value of human life.

## 3. Results

### 3.1. Demographic and Professional Characteristics

The demographic and professional characteristics of the 71 physicians/participants are presented in Table 1. The majority of our study participants were women (76.8%), 30–39 years of age (49.3%), with children (55.1%), who considered the role of religion in their life important (69.6%). The majority of physicians worked in university-affiliated hospitals (63.8%), lived in rural areas (66.7%) and had a professional experience in NICU for up to 15 years (85.5%). The respective characteristics of the entire sample of the study, i.e., 251 healthcare professionals, as well as the attributes of NICUs pertaining to equipment and staff are detailed in Daglas, Petousi & Poulios [50].

### 3.2. Physicians’ Self-Reported Practices

Physicians were initially asked whether they had ever taken a decision (on their own or jointly with others) to limit intensive care to a newborn who was close to death or had an unfavorable prognosis (Table 2). Of the total sample, 37.7% responded that they had limited intensive care in one or more cases of infants close to death and 30.8% that they had done so in one or more cases of infants with unfavorable neurological prognosis. A large percentage of physicians (42%) responded “maybe” to the question of whether they had limited provision of intensive care to a neonate close to death (17.4%) or with an unfavorable prognosis (24.6%) indicating a reluctance or “cautiousness” in their response (Table 2).

### 3.3. Factors Related to Ethical Decision Making

The exploration of the factors related to ethical decision making indicates that physicians who had taken a decision to limit intensive care in newborns close to death compared to physicians who had not taken such a decision, tend to work in NICUs with a higher average number of mechanical ventilation devices (respirators) (*p* = 0.045) and midwives and nurses (*p* = 0.016) (Table 3). Also, this self-reported attitude of physicians seems to be linked to their attitude towards the value of human life. Physicians who reported that they had taken such a decision, on average, have a higher “attitude score” than the rest of the physicians (*p* = 0.026). It follows that these physicians tend to ascribe to the quality of life approach (Table 3). Additionally, our study shows that professional experience is a factor that affects ethical decision-making in neonatal intensive care. Specifically, physicians who report that they had taken the decision to limit intensive care in newborns with unfavorable neurological prognosis, on average, had more years of experience than the rest of the physicians (*p* = 0.005) (Table 3).

According to our logistic regression analysis model, two of the prediction variables were found to impact on Greek physicians’ self-reported participation in decisions regarding the limitation of intensive neonatal care (Table 4). Specifically, physicians who are against the involvement of parents in the decision-making process concerning treatment of their children, are dramatically less probable than their counterparts, to report that they have taken such decision (*p* < 0.05) (Table 4). At the same time, physicians with higher “attitude scores” have a higher probability of reporting that they have taken a decision to limit neonatal intensive care (*p* < 0.05). In other words, the higher the support of Greek physicians for the value of quality of human life, the more probable it is that they have decided to withhold or withdraw neonatal intensive care (Table 4).

### 3.4. The Most Common Ethical Decisions

**Figure 1:**
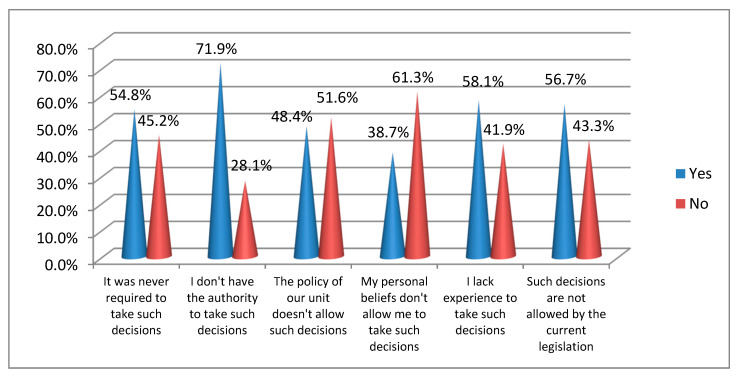
Main reasons that prevented neonatologists to take an ethical decision during their career.

As Greek neonatologists reported in this study, the most common ethical decisions that were taken by them involved mostly withholding of intensive care, i.e., continuation of the current treatment without addition of any other treatment (73.9%) and non-implementation of urgent treatment (58.3%) (Table 5). Decisions regarding feeding of newborns, e.g., no application of feeding tube and parenteral nutrition, have rarely been taken. Moreover, only one participant reported that they had administered lethal medication to a hospitalized infant once in their career (Table 5). The main reasons that have prevented physicians in our sample to take an ethical decision is the lack of authority (71.9%) and experience (58.1%) to take such decisions as well as the existing Greek legislation (56.7%) (Figure 1).

## 4. Discussion

The goal of the present study was to document self-reported practices and involvement of Greek neonatologists serving in NICUs in the face of ethical dilemmas related to neonatal intensive care as well as the factors affecting these practices and involvement. Collection of data was based on the well-respected, frequently implemented EURONIC project questionnaire. Thus, our findings can be compared to the findings of studies that have been conducted in other countries. Our study’s main findings, which we discuss in the following sections, relate to: the proportion of neonatologists admitting to limiting neonatal intensive care, the practices they are most likely to implement in such instances and the factors that are more likely to influence these practices. Findings of similar studies in other European countries, Australia and New Zealand [21,52,53,54], as well as the overall contention of the EURONIC project indicate that a large proportion of neonatologists in European countries admit that they have limited the provision of intensive care to newborns close to death at least once in their career [21]. Our data, however, show that approximately one-third of the neonatologists in our sample concede to having at least once in the past (either alone on jointly with others) limited intensive care a) to a newborn close to death (37.7%) or/and b) to a newborn with unfavorable neurological prognosis (30.8%).

There are several possible explanations for these findings. First off, Greece is lacking a framework regulating the withdrawal or withholding of intensive care and euthanasia overall. Studies, however, reveal the importance of the legal framework and show that physicians’ practices are influenced by it [36]. However, as mentioned above, Greece at the time the study took place as well as today is lacking such a framework. Thus, Greek neonatologists who limit neonatal intensive care risk facing serious charges (art. 300 of the Penal Code on homicide and/or participation in homicide) and severe penalties with potentially detrimental impact on their personal lives and professional careers. In light of this, it is plausible that Greek neonatologists will be reluctant to either limit intensive care or admit to having done so. Besides, Greek neonatologists’ reluctance or “cautiousness” in admitting involvement in withholding/withdrawing neonatal intensive care is further evidenced by the finding that they neither confirm nor reject their participation in withholding/withdrawing neonatal intensive care either in newborns close to death (17.4%) or in newborns with unfavorable prognosis (24.6%). Along these lines it is worth noting that a large proportion of our sample reported that they “do not have the authority to make such decisions” pointing probably to a tendency to transpose responsibility for such decisions. Nevertheless, the proportion of neonatologists in our sample who respond “maybe” to the relevant question far exceeds the proportion of equivalent responses in other studies at the European level [21]. Although not empirically tested in the present study, Greek neonatologists documented “cautiousness” may further attest to the significance of the legal framework or lack thereof, and consequently, explain the reported low level of relevant decision-making.

An additional possible explanation for the small proportion of Greek neonatologists who admit withholding and/or withdrawing intensive care is the finding that Greek healthcare professionals in NICUs hold strong vitalistic attitudes [50]. Greek neonatologists, that is, ascribe to the principle of the intrinsic value of human life in rates much higher than the equivalent rates reported in other studies in Europe and worldwide [39,50,52,53,55]. It is likely then, that this attitude further influences their practices (or admission thereof) concerning limits to neonatal intensive care.

Greek neonatologist’s low admission rates of limitation to intensive care may further be linked to factors related to their status and perceived role as professionals. Peng et al. [56] for example, in their study, have found that healthcare professionals in neonatal care in Taiwan report feelings of “failure” and inability to fulfil their professional role when they are unable to maintain human life. Similarly to their colleagues in Taiwan, Greek physicians are accorded high social status, prestige and authoritative knowledge [57]. At the same time, the medical model in Greece continues to be mainly paternalistic despite indications of change [58,59,60]. In this context, it may be the case that Greek neonatologists in NICUs persist with provision of intensive care to neonates because they perceive this to be part of their professional role and obligations.

Even if rarely, Greek neonatologists admit that in the face of ethical dilemmas concerning neonatal intensive care they are more likely to withhold rather than withdraw care. Specifically, they report continuation of the current treatment without addition of any other (73.9%) or no application of an urgent treatment (58.3%). This is in line with the findings of other studies that report the practice of withholding as prevailing over the practice of withdrawing intensive care. This holds true for European countries in general [38,61] and even more so for Central and Southern European ones [21]. Moreover, the finding that Greek neonatologists tend to withhold rather than withdraw intensive care aligns with findings of the European study ETHICUS [62]. The study addressed issues of adult intensive care provision and found that physicians tended to withhold rather than withdraw it [44]. It may be the case then, that implementation of similar practices in the different contexts of adult and neonatal intensive care by Greek physicians, particularly in light of a missing regulatory framework, is influenced by cultural characteristics that may serve as points of reference during the decision-making process.

Although withholding is prioritized over withdrawing of intensive care, our study shows that withholding feeding and hydration of newborns is rarely implemented. This concurs with findings of studies concerning treatment of neonates in other countries [63]. It also concurs with findings of studies concerning the treatment of adults in Greece. Tsaloglidou et al. [64] for example, report that limitation of food and water raises significant dilemmas for the scientific community. Moreover, they assert that these are the most difficult decisions to take in the context of end-of-life care. Food and water, even when provided through mechanical means, represent the main link to human existence and life. Therefore, limiting their provision leads to certain death of the patient [64] and thus, it can be equated to withdrawal of care.

Notwithstanding the type of limitations of intensive care, our study shows that neonatologists in Greek NICUs are more likely to implement them in the case of infants close to death and less so, to neonates with an unfavorable neurological prognosis. Similarly, the “cautiousness” of Greek NICU physicians discussed earlier, is more likely to be expressed with respect to neonates with unfavorable prognosis rather to infants close to death. Greek neonatologists’ practices are comparable to the practices of their counterparts in countries such as Italy [21], Turkey [52] and Ireland [53] as shown in relevant, similar studies. For example, the percentage of Greek physicians who have decided to limit life supportive interventions in newborns with unfavorable neurological prognosis, is similar to the equivalent of their Italian colleagues [21]. What is of importance to note is that the above mentioned practices of Greek NICU neonatologists correspond to practices of their counterparts in counties such as Italy, Ireland and Turkey that tend to exhibit unifying characteristics such as a prevalent religious affiliation and/or strong identification with the respective religion or dogma. In turn, this observation points to the importance various factors including socio-cultural and demographic characteristics as well as the value system of physicians play in shaping their ethical decision-making.

Logistic regression analysis aiming at identifying factors associated with physicians’ decisions to withdraw or withhold neonatal intensive care, revealed a relation between physicians’ attitude towards the involvement of parents in decisions related to the care of neonates and physicians’ likelihood to have taken such a decision. Specifically, physicians opposing parental involvement were less likely to report having taken a decision to withdraw or withhold intensive care. Reversely, physicians supportive of parents’ involvement were more likely to report relevant decisions. The aetiology of the above finding cannot be empirically assessed in the context of the present research. It is, however, an important finding that merits further attention and examination in light of changes in the medical practice towards more active engagement and involvement of users of medical services.

Physicians’ attitude towards the value of human life was a second factor found to have an impact on their likelihood to decide over withdrawing or withholding intensive care. Specifically, physicians who tend to attribute higher value to the quality of human life (see measurements and scale in Daglas, Petousi & Poulios [50]) are more likely than their counterparts who side with the argument of life’s intrinsic value to report that they have taken a decision to limit provision of intensive care. The statistical significance of the attitude of physicians towards the value of human life and its impact on the clinical practices of physicians have been corroborated by findings in the EURONIC project and other research as well [39,52,53].

In other studies, Guimarães et al. [38] and Abdel Razeq [35] for example, additional factors predictive of physicians’ practices have been identified; prominent among them are cultural and religious factors. Although religion was not revealed as a statistically significant variable predicting physicians’ decision-making when faced with ethical dilemmas in neonatal intensive care, its relevance cannot be ignored in the context of the present study. First off, as reported elsewhere [50], Greek neonatologists’ attitude towards the value of human life depends on the role that religion plays in their life; a finding similar to that of the EURONIC project [39]. Moreover, findings of the ETHICUS project, which focused on decision-making in the context of end-of-life care, point to a similar pattern for Greek physicians. It is concluded in the aforementioned project that there exists a complex interrelationship between religion and other cultural characteristics such that it is not always clear to distinguish their individual effects on people’s behavior. On the other hand, there are instances in which their effect impacts on behavioral patterns. For example, project ETHICUS reports that Greek Orthodox and Jewish physicians reported a much lower degree of withdrawing of intensive care compared to the rest of the participants from other countries and religions [44].

Findings reported in the present article are based exclusively on data from self-reported practices of physicians working in Greek NICUs. Corroborating data from NICU records were not available. This represents a limitation of the present study taking into consideration the potential that social desirability bias (respondents’ tendency to underreport activities and/or practices), which are either considered, or respondents perceived, as being illegal and/or socially undesirable and overreport socially desirable activities and/or practices, may distort estimates [65]. Nevertheless, surveys based on self-reported practices have been repeatedly used for the evaluation of ethical dilemmas and many international studies have been based on these practices [21,52,53]. Moreover, the international literature and practice shows that the impact of the social desirability bias can be reduced by carefully planning data collection strategies and overall reduce respondents’ discomfort when answering sensitive questions including the discomfort caused by the potential of personal identification [65]. As it is true for any study, we cannot entirely rule out the potential that some of our participants framed their responses in social desirability terms and/or in some reservation over anonymity. We have, however, been diligent in the implementation of informed consent procedures. Potential participants were fully informed both orally and in writing as to the purpose of the study and its methodology. Particular attention was given to informing participants on the way the principles of confidentiality were to be respected and implemented throughout the study. The relevant procedures have been detailed in another publication [48].

A second possible limitation of the present study is the fact that data were collected some time ago. However, since the time of data collection, no regulatory changes that could potentially impact on our findings have occurred. Specifically, there are no changes in the legal framework. Greece continues to lack a legislative and overall regulatory framework concerning withholding and/or withdrawing of intensive care for neonates and adults alike. Similarly, no biding guidelines framing the provision of neonatal intensive care have been implemented. Nonetheless, in 2018 and in the context of harmonizing Greek perinatal data reporting to the EU standards, the Greek Ministry of Health has issued a Ministerial Decree (Pr. No. 82821/29-10-2018) which a) defines the limit of viability at week 22 of gestation, and b) requires that all stillbirths from week 22 and beyond are recorded and reported. Consequently, practices and protocols of neonatal intensive care continue to be defined at the level of each NICU.

Finally, a limitation of the present study relates to the gender distribution within our sample. The number of men in our sample is considerably lower than the number of women. Thus, we were not able to perform reliable statistical analysis to investigate potential gender differences. However, the gender imbalance in our sample is a function of the gender distribution of NICU physicians; a considerably higher number of women compared to men are employed full time in the NICUs included in our sample. This imbalance could probably be corrected if we had implemented another sampling approach targeting the inclusion of more men participants. Nevertheless, given the sensitivity of the research topic, we limited our direct contact with potential participants as an additional safeguard of confidentiality and non-identification.

Significant, however, are the contributions of the present study. Firstly, to the best of our knowledge, this is the first study ever to explore the practices of ethical decision-making by Greek physicians concerning intensive care of preterm and ill newborns. Moreover, the study provides data for all 17 public NICUs in the country, thus covering a wide geographical area and accounting for differences in the infrastructure and operation of the different units. An additional contribution of the study stems from the use of a standardized, internationally implemented instrument, the EURONIC questionnaire adjusted to the needs of the current research and the specificities of the country [48]. This way, data on Greek neonatologists’ ethical decision-making practices can be compared to the findings of other equivalent studies in other countries and health care systems and further contribute to the international scholarship.

## 5. Conclusions

This study delved into the determinants of the decision-making process of Greek neonatologists and placed emphasis on decisions to limit provision of intensive care. Our study showed that a relatively small proportion of Greek neonatologists admit to having implemented limits to neonatal intensive care. Mainly, Greek neonatologists’ decision-making is determined by their socio-cultural characteristics and their value system, their tendency to align with the principle of the inherent value of life rather than the principle of the quality of life. Together with other international studies [21,37,38,39,66], our study shows that there are significant differences in the implementation of limits in interventional medical practices during the provision of intensive neonatal care. In light of the rapidly advancing biomedical and technological advances, it is probable that health care professionals will be faced with increasingly new and serious bioethical dilemmas. Therefore, the factors that influence and determine the ethical decision-making of health care professionals in the face of such dilemmas need to be comprehensibly understood in order to safeguard quality of care and respect for human life. Further exploration of these factors is required in order to properly deal with bioethical dilemmas in NICUs.

## Figures and Tables

**Table 1:** Demographic and professional characteristics of neonatologists.

Demographic Characteristic	*n*	*%*
**Age**		
<30 years	4	5.8
30–39 years	34	49.3
40–49 years	19	27.5
50 years and over	12	17.4
**Gender**		
Male	16	23.2
Female	53	76.8
**Having Children**		
Yes	38	55.1
No	31	44.9
**Importance of Religion**		
Important	48	69.6
Not important	21	30.4
**Professional Characteristic**		
**Institution**		
University Hospital	44	63.8
Public Hospital	25	36.2
**Hospital Location**		
Urban	23	33.3
Rural	46	66.7
**Experience at NICU**		
<6 years	43	62.3
6–15 years	16	23.2
>15 years	10	14.5

**NICU:** Neonatal Intensive Care Unit.

**Table 2:** Physicians’ self-reported practices throughout their professional life in neonatal intensive care.

Have You Ever Decided (Alone or Together with Others) to Set Limits to Intensive Interventions? Because of:	Yes *f* (*f*%)	Maybe *f* (*f*%)	No *f* (*f*%)
infant’s fatal/terminal condition	26 (37.7%)	12 (17.4%)	31 (44.9%)
particularly poor neurological prognosis	20 (30.8%)	16 (24.6%)	29 (44.6%)

**Table 3:** The influence of the NICU “environment” (or the endogenous characteristics of NICUs) and the attitude score for the value of life in self-reported decisions of physicians.

**Physicians Who Have Ever Decided to Set Limits to Intensive Interventions Because of Infant’s Fatal/Terminal Condition:**							
**Variables**	**Yes**		**Maybe**		**No**		***p***
	**Mean**	**Std. Deviation**	**Mean**	**Std. Deviation**	**Mean**	**Std. Deviation**	
Number of mechanical respirators	11.7 (10.1–13.4)	3.7	9.8 (7.4–12.3)	3.6	8.9 (7.4–10.4)	4.8	**0.045**
Number of nursing neonates	33.1 (28.8–37.3)	11.4	28.8 (22.6–35.1)	10.6	28.7 (24.8–32.6)	10.5	0.28
Number of physicians	10.6 (9.3–11.9)	3.7	9.3 (7.4–11.1)	2.7	10.1 (8.9–11.2)	3	0.508
Number of midwives and nurses	23.4 (21.1–25.7)	6.2	18.9 (15.5–22.3)	6.4	19.1 (17–21.2)	5.4	**0.016**
Total number of employees	34 (30.7–37.2)	8.5	28.2 (23.4–32.9)	8.6	29.1 (26.2–32.1)	8	0.051
Professional experience at NICU (months)	110.7 (73.2–148.3)	94.4	102.5 (46–159.1)	87.1	54.6 (20.3–88.8)	95.7	0.075
Attitude score	24.7	3	23.5	5.6	21.4	4.3	**0.026**
**Physicians Who Have Ever Decided to Set Limits to Intensive Interventions Because of Extremely Poor Neurological Prognosis:**							
**Variables**	**Yes**		**Maybe**		**No**		***p***
	**Mean**	**Std. Deviation**	**Mean**	**Std. Deviation**	**Mean**	**Std. Deviation**	
Number of mechanical respirators	10.2 (8.3–12.1)	3	10.5 (8.3–12.7)	3.9	9.6 (8–11.2)	5.3	0.773
Number of nursing neonates	30.3 (25.3–35.3)	10.7	28.4 (22.9–34)	11.1	30.7 (26.5–34.8)	11.3	0.806
Number of physicians	9.6 (8.1–11)	3.2	10.1 (8.5–11.8)	3.9	10.2 (9–11.4)	3.1	0.766
Number of midwives and nurses	21.6 (18.9–24.3)	6.3	19 (15.9–22.1)	6.3	20.4 (18.2–22.7)	5.9	0.454
Total number of employees	31.2 (27.3-35)	8	29.1 (24.8–33.4)	9.2	30.7 (27.5–33.9)	8.6	0.766
Professional experience at NICU (months)	117.6 (79.5–155.6)	101.4	93.4 (48–138.9)	91.6	36.5 (4.4–68.6)	67.2	**0.005**
Attitude score	25.1	2	22.2	5.8	22.3	4.1	0.054

**NICU:** Neonatal Intensive Care Unit.

**Table 4:** Logistic regression analysis for the statistical prediction of decision-making regarding the limitation of neonatal intensive care by physicians.

Prediction Variables	*Β*	*Wald χ^2^*	*Odds Ratio*	*Confidence Internal*
Gender (man)	0.22	0.06	1.24	0.20–7.66
Age (<40)	0.24	0.06	1.27	0.18–8.87
Experience at NICU	0.01	3.49	1.01	1.00–1.03
Importance of religion (no)	0.40	0.22	1.49	0.28–7.87
Having children (no)	−0.60	0.72	0.55	0.14–2.18
**Health care professionals’ agreement with parents’ involvement during the bioethical decision-making process (no)**	**−0.98**	1.81 *	**0.37**	**0.09–1.57**
**Attitude score on the value of human life**	**0.22**	5.92 *	**1.24**	**1.04–1.48**

Note. * *p* < 0.05. Dependent variable: The bioethical decision taken by neonatologists to limit the intensive care of a newborn Close to death or a newborn with unfavorable neurological prognosis. *Nagelkerke R^2^ =* 0.06, *χ*^2^ (7, 71) = 16.21, *p* < 0.05. **NICU:** Neonatal Intensive Care Unit.

**Table 5:** Bioethical decisions taken by neonatologists for the limitation of neonatal intensive care in Greece.

Neonatologist Had Decided:	Yes, More Than Once f% (f)	Yes, Only Once f% (f)	Never Decided f% (f)
Continuation of current treatment **without** addition of any other treatment	73.9% (34)	10.9% (5)	15.2% (7)
**Non** implementation of urgent treatment/handling (e.g., cardiopulmonary resuscitation)	58.3% (28)	6.3% (3)	35.4% (17)
**Avoid** changing respirator parameters	42.6% (20)	8.5% (4)	48.9% (23)
**Non** starting of intensive care (e.g., resuscitation at birth, mechanical ventilation)	37.5% (18)	14.6% (7)	47.9% (23)
**Termination** of mechanical ventilation	29.2% (14)	10.4% (5)	60.4% (29)
**Termination** of life-saving medication (e.g., cardiotonic medication)	25.5% (12)	6.4% (3)	68.1% (32)
**Administration** of tranquillizers and/or analgesics for the relief of pain, although this may lead to respiratory depression and death	25.5% (12)	6.4% (3)	68.1% (32)
**Non** administration of antibiotics	13% (6)	15.2% (7)	71.7% (33)
**No** surgery	12.8% (6)	14.9% (7)	72.3% (34)
**No** application of full parenteral nutrition	8.5% (4)	8.5% (4)	83% (39)
**No** application of feeding tube	4.3% (2)	2.2% (1)	93.5% (43)
**Administration** of medication for the termination of the life of the newborn/infant	0% (0)	2.1% (1)	97.9% (47)

Note: Only the answers of physicians who replied either “yes” or “maybe” to the question “Have you ever decided (alone or together with others) to set limits to intensive interventions?” are reported in this table.
